# Respiratory drugs and psychiatric adverse events in children and adolescents: a pharmacovigilance study based on the FAERS database

**DOI:** 10.1007/s00210-026-05075-5

**Published:** 2026-02-06

**Authors:** Jing Feng, Shujuan Zhao

**Affiliations:** Department of Pharmacy, Henan Provincial People’s Hospital, People’s Hospital of Zhengzhou University, School of Clinical Medicine, Henan University, Zhengzhou, 450003 Henan China

**Keywords:** Respiratory drugs, Psychiatric, Adverse events, Pediatric, FAERS

## Abstract

The association between respiratory drugs (RDs) and pediatric psychiatric adverse events (pAEs) remains insufficiently characterized. We aimed to characterize pAEs associated with RDs use in pediatric patients. RD-related pAE reports were retrieved from the U.S. Food and Drug Administration Adverse Event Reporting System (FAERS) database. Analysis focused on pediatric patients aged 0 to 17 years. Disproportionality analysis was conducted using four algorithms, with a focus on anxiety disorders and symptoms, depressed mood disorders and disturbances, and suicidal and self-injurious behaviors. Descriptive and clinical outcome analyses were performed, along with subgroup analyses to explore potential risk differentials by age and gender. Among 6,994 cases of RD-related pAEs in pediatric patients, 16 drugs with positive signals were identified across all four algorithms, with antiallergics accounting for 75.00%. Montelukast was the most frequently reported drug (*n* = 6,168), primarily associated with anxiety (21.84%, reporting odds ratio [ROR] = 15.08), depression (15.86%, ROR = 12.90), and suicidal ideation (14.27%, ROR = 13.63). Promethazine demonstrated strong signals for intentional self-injury (62.99%, ROR = 127.36). The highest mortality rates were observed with hydroxyzine (50.94%) and caffeine (50.00%), while oxytetracycline (4/4, 100%) showed the highest proportions of life-threatening events. Montelukast generated signals in all age and gender groups, with the highest disproportionality observed in children aged 0 to 4 years. This study identifies statistical associations between RDs and pAEs in pediatric patients, particularly with montelukast and promethazine. The highest mortality rates were observed with hydroxyzine. These findings provide a foundation for further research but require confirmation through large-scale prospective studies.

**Supplementary Information:**

The online version contains supplementary material available at 10.1007/s00210-026-05075-5.

## Introduction

Respiratory drugs (RDs) are among the most frequently prescribed agents in pediatric practice due to the high prevalence of respiratory conditions in children and adolescents (Leporini et al. 2022). These medications play a crucial role in managing common childhood conditions such as asthma, allergic rhinitis, and respiratory infections. However, despite their widespread use, the safety profiles of RDs, particularly regarding psychiatric adverse events (pAEs), remain incompletely characterized in pediatric populations.

Drug safety in children requires special consideration due to developmental differences in pharmacokinetics, pharmacodynamics, and organ systems. Children and adolescents are in a critical period of brain development, their developing nervous systems may exhibit heightened sensitivity to neuropharmacological effects. This heightened sensitivity can result in more severe psychiatric symptoms compared to adults. Therefore, pAEs potentially associated with drug use are a critical consideration in pediatric therapeutics, as exemplified by the psychiatric adverse events reported with montelukast use (Bian et al. 2021).

Pharmacovigilance research provides a valuable framework for identifying and characterizing rare but serious adverse events (AEs) (Lavertu et al. 2021). The U.S. Food and Drug Administration (FDA)'s Adverse Event Reporting System (FAERS), one of the largest spontaneous reporting systems globally, serves as a powerful platform for signal detection and the evaluation of AEs in real-world clinical practice (U.S. Food and Drug Administration 2024a). Such spontaneous reporting systems allow for the systematic analysis of AE patterns, offering insights into the relationship between drug exposure and pAEs that may not be apparent in short-term clinical trials.

Recent epidemiological data indicate a concerning prevalence of psychiatric disorders in pediatric populations. A meta-analysis (Polanczyk et al. 2015) synthesizing data from 41 studies conducted across 27 countries reported a 13.4% overall prevalence of psychiatric disorders in individuals under 18 years. Anxiety and depressive disorders constituted significant proportions, with prevalence rates of 6.5% and 2.6%, respectively. Meanwhile, suicide remains a critical global health challenge, responsible for approximately 800,000 deaths annually and accounting for 1.3% of worldwide mortality (Sher and Oquendo 2023). Given these patterns, our study specifically focuses on signals of anxiety disorders and symptoms, depressed mood disorders and disturbances, and suicidal and self-injurious behaviors associated with RDs.

Using the FAERS database, we aimed to: (1) Identify signals of RD-related pAEs in pediatric patients through disproportionality analyses. (2) Characterize the specific types and clinical outcomes of RD-related pAEs. (3) Conduct a comparative analysis of signals across age and gender groups.

## Methods

###  Data source and case selection

This study utilized a pharmacovigilance approach based on the FAERS database, a publicly accessible repository of safety reports submitted by patients, healthcare professionals, and pharmaceutical companies (U.S. Food and Drug Administration 2023). FAERS data files from Q1 2004 to Q3 2024 were downloaded for analysis.

The study population was defined by the following criteria: (1) Pediatric patients aged 0 to 17 years. (2) RDs coded as “primary suspected (PS)” to focus on the most probable drug-event associations. RDs were identified based on the World Health Organization's Anatomical Therapeutic Chemical (WHO ATC) classification system (R category), including nasal preparations, throat preparations, drugs for obstructive airway diseases, cough and cold preparations, antihistamines for systemic use, and other respiratory system products. While all RDs reported in FAERS were initially analyzed, only those generating signals across all four disproportionality algorithms are presented in Results. (3) Adverse events classified under the “psychiatric disorders” System Organ Class (SOC) (code 10,037,175) according to the Medical Dictionary for Regulatory Activities (MedDRA, version 27.1) (Medical Dictionary for Regulatory Activities 2022). We specifically focused on three high-level group terms (HLGTs): anxiety disorders and symptoms (code 10,002,861), depressed mood disorders and disturbances (code 10,012,375), and suicidal and self-injurious behaviors (code 10042460).

MedDRA is an international standard for pharmacovigilance terminology, which ensures the consistency in AE reporting across regulatory activities. This dictionary is arranged into five distinct hierarchical levels: low-level terms (LLTs), preferred terms (PTs), high-level terms (HLTs), HLGTs, and SOCs.

**Fig. 1:**
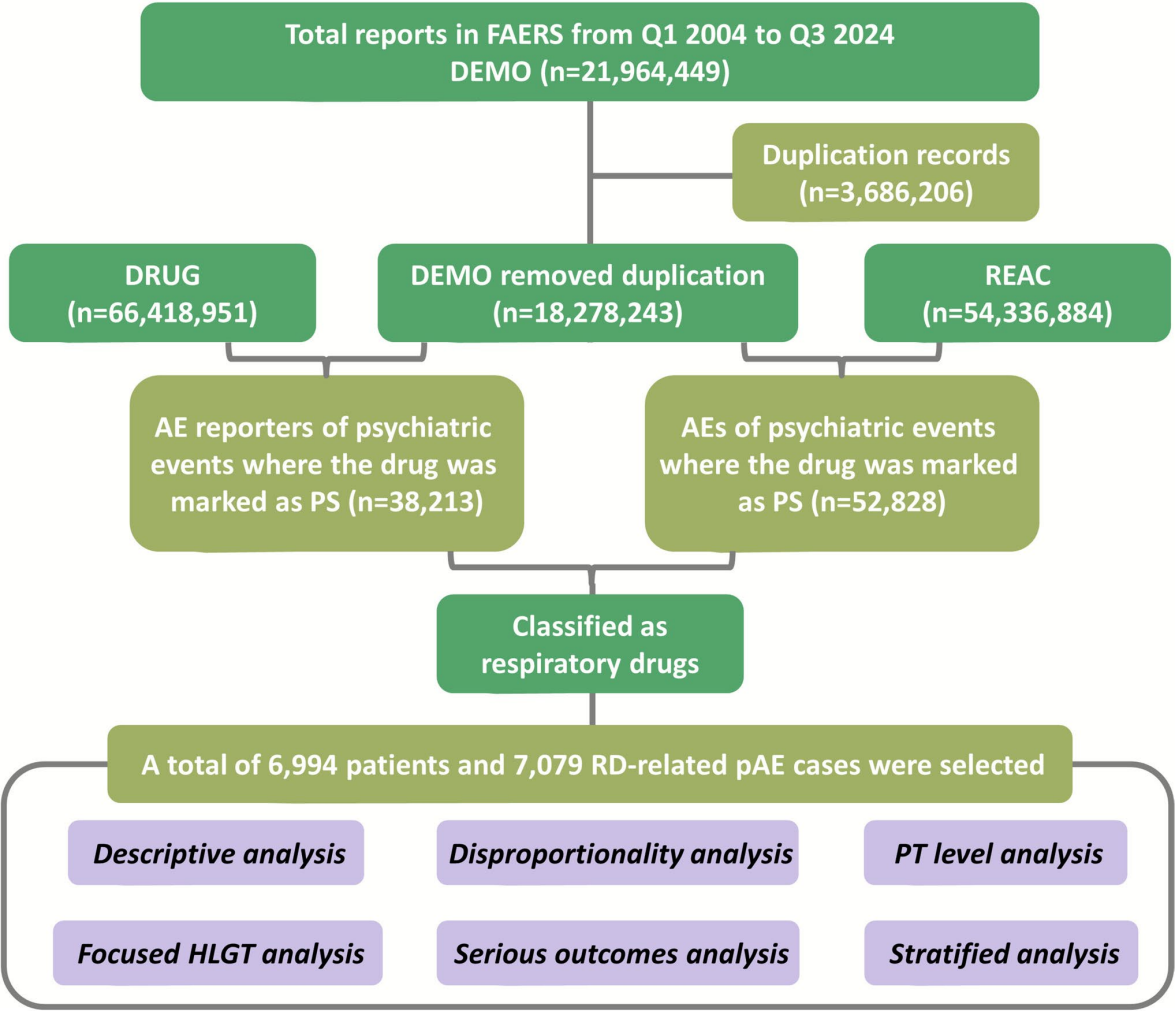
The flowchart illustrating the comprehensive study process. Abbreviations: DEMO, demographic and administrative information; DRUG, drug specifics; REAC, adverse events; AE, adverse event; PS, primary suspect; pAE, psychiatric adverse event; PT; preferred term; HLGT, high level group term

In this study, PTs represent distinct descriptors for single medical concepts (e.g., symptoms, signs, disease diagnoses, therapeutic indications, or medical history characteristics) (Lu et al. 2025). HLGTs are utilized to provide a broader overview of potential adverse event patterns. Following these criteria, a total of 6,994 patients and 7,079 RD-related pAE reports in pediatric populations were identified and included for analysis (Fig. 1).

###  Data processing procedure

Following FDA guidelines (U.S. Food and Drug Administration 2024b), a deduplication process was conducted to ensure the accuracy and reliability of the AE reports. During this process, only the most current version of each case was retained, while all duplicate versions were removed. Specifically, the PRIMARYID, CASEID, and FDA_DT fields were extracted from the DEMO data file. The data were then sorted in the following order: CASEID, FDA_DT, and PRIMARYID. For cases with multiple reports sharing the same CASEID, the report with the most recent FDA_DT value was retained. In instances where multiple reports had identical CASEID and FDA_DT values, the report with the highest PRIMARYID was retained. Additionally, starting from the first quarter of 2019, each quarterly FAERS data package has included a list of deleted reports, which we used to remove corresponding entries after deduplication. This process ensured that only active and valid reports were included for subsequent analysis.

###  Descriptive analysis

A descriptive analysis was conducted to summarize the clinical characteristics of reports involving RD-related pAEs after screening. The variables analyzed included gender, age, reporter type, reported countries, route of administration, therapeutic indication, clinical outcome, AE occurrence time, and reporting year. Reports classified as having serious outcomes included life-threatening events, hospitalization, disability, death, congenital anomalies, required intervention, and other serious medical events. The occurrence time of RD-related pAEs was calculated by subtracting the therapy start date from the event start date.

###  Disproportionality analysis

In pharmacovigilance studies, disproportionality analysis is a key method for evaluating possible associations between specific AEs and particular drugs. Signal detection was performed using a case/non-case approach based on a 2 × 2 contingency table, where cases were defined as reports with the target respiratory drug and psychiatric disorders, and non-cases included all other drug-adverse event combinations. A minimum of three reports was required for signal calculation. No adjustment for multiple comparisons was applied, as this study was designed as an exploratory investigation. Beyond statistical analysis, we evaluated the clinical plausibility of identified drug-event pairs based on known pharmacological properties and existing literature.

In this study, four methods were employed to detect adverse drug event signals (Bate et al. 1998; Rothman et al. 2004; Moore et al. 2005; Sakaeda et al. 2013): reporting odds ratio (ROR) method; proportional reporting ratio (PRR) method, which detects signals based on the lower limit of the PRR confidence interval; the British Medicines and Healthcare Products Regulatory Agency (MHRA) criterion, a variation of the PRR method with different threshold settings; and Bayesian Confidence Propagation Neural Network (BCPNN) method. The details of these methods are provided in Table S1 and Table S2. For this study, a psychiatric adverse reaction signal was considered valid and associated with RD treatment only when the criteria for all four algorithms were met simultaneously.

The disproportionality analysis was performed in several steps: (1) Evaluation of RD-related pAEs was conducted using four algorithms. (2) The frequency and intensity of RD-related pAE signals were analyzed at the PT level. (3) Given the large volume of reported cases for montelukast, a detailed disproportionality analysis of pAEs associated with this drug was performed at the PT level. (4) Related AEs were grouped and analyzed at the HLGT level, including anxiety disorders and symptoms, depressed mood disorders and disturbances, and suicidal and self-injurious behaviors. (5) Cases involving death and life-threatening events were compared to assess the severity of RD-related pAEs. (6) To explore potential differences in the relationship between RDs and pAEs, stratified analyses were conducted by age and gender.

It should be emphasized that disproportionality signals indicate statistical associations between drugs and AEs but cannot establish causality. These findings require confirmation through controlled epidemiological studies or clinical trials.

###  Statistical analyses

All statistical analyses and visualizations were performed using SPSS version 27.0 (IBM Corp., Armonk, NY, USA), R software version 4.4.2 (R Foundation for Statistical Computing, Vienna, Austria), and the Prism version 9.5 (GraphPad Software, San Diego, CA) software package.

###  Reporting guidelines and ethical statement

This pharmacovigilance study was conducted in accordance with the REporting of A Disproportionality Analysis for DrUg Safety Signal Detection Using Individual Case Safety Reports in PharmacoVigilance (READUS-PV) guidelines (Fusaroli et al. 2024).

This study utilized data from the publicly accessible FAERS database, where all patient records are anonymized and de-identified. Therefore, ethical clearance and informed consent were not required for this research.

## Results

###  Descriptive analysis

From Q1 2004 to Q3 2024, a total of 6,994 pediatric patients were documented in the FAERS database (Table 1). Excluding cases with missing data (*n* = 146, 2.09%), the gender distribution was nearly balanced, with females accounting for 49.23% (*n* = 3,443) and males for 48.68% (*n* = 3,405). Patients were divided into three age groups. Adolescents aged 12–17 years accounted for the highest proportion of reports (*n* = 3,225, 46.11%), followed by children aged 5–11 years (*n* = 2,731, 39.05%) and infants/toddlers aged 0–4 years (*n* = 1,038, 14.84%). Reports were primarily submitted by healthcare professionals (physicians, pharmacists, and other health professionals), who contributed 54.28% of cases, while consumers accounted for 38.39% of submissions. Geographically, the majority of reports originated from the United States (54.73%). Among the RDs involved, the most common route of administration was oral (61.45%), and the most frequently reported therapeutic indication was asthma (28.02%). Notably, 82.59% (*n* = 5,776) of the reports were classified as serious, with “other serious outcomes” being the most frequently reported category (59.44%), followed by hospitalization (24.64%), disability (9.27%), life-threatening events (8.64%), and death (5.82%). The median time to pAE onset was 4.5 days (interquartile range [IQR]: 0–136.0). Furthermore, there was a marked upward trend in pAE reports in recent years, with 50.74% of cases reported during the 2019–2024 period.

**Table 1:** Characteristics of RD-related pAE reports in pediatric patients

Characteristics	Number of patients (*n*, %)
Overall	6,994 (100.00)
Gender	
- Female	3,443 (49.23)
- Male	3,405 (48.68)
- Not specified	146 (2.09)
Age (years)	
- Median (IQR)	11.0 (6.0–15.0)
- 0–4 years	1,038 (14.84)
- 5–11 years	2,731 (39.05)
- 12–17 years	3,225 (46.11)
Reporter	
- Consumer	2,685 (38.39)
- Physician	1,913 (27.35)
- Other health-professional	986 (14.10)
- Pharmacist	897 (12.83)
- Not specified	497 (7.11)
- Lawyer	16 (0.23)
Reported countries (The top 3)	
- United States	3,828 (54.73)
- United Kingdom	1,112 (15.90)
- Sweden	317 (4.53)
Route	
- Oral	4,298 (61.45)
- Not specified	1,699 (24.29)
- Subcutaneous	578 (8.26)
- Respiratory (Inhalation)	294 (4.20)
Indication	
- Asthma	1960 (28.02)
- Not Specified	2099 (30.01)
- Dermatitis atopic	296 (4.23)
- Cystic fibrosis	293 (4.19)
Degree	
- Serious	5,776 (82.59)
- Non-serious	1,218 (17.41)
Outcomes^#^	
- Life-threatening	604 (8.64)
- Hospitalization (Initial or prolonged)	1,723 (24.64)
- Disability	648 (9.27)
- Death	407 (5.82)
- Congenital anomaly	13 (0.19)
- Required intervention	116 (1.66)
- Other	4,157 (59.44)
AE occurrence time, days	
- Median (IQR)	4.5 (0–136.0)
- 0–7 d	1,949 (27.87)
- 8–60 d	351 (5.02)
- 61–180 d	818 (11.70)
- 181–360 d	3,876 (55.42)
Reporting year	
- 2004–2008	904 (12.91)
- 2009–2013	1,215 (17.35)
- 2014–2018	1,331 (19.00)
- 2019–2024	3,544(50.74)

^#^: Some report cases generated > 1 adverse event outcomes

Abbreviations: *RD* Respiratory drug, *pAE* Psychiatric adverse event, *IQR* Interquartile range

###  Identification of drug signals using disproportionality analysis

To evaluate the potential risk of RD-related pAEs in pediatric patients, disproportionality analysis was conducted. Signal detection was performed at the drug level, assessing the association between each respiratory drug and the “psychiatric disorders” SOC. A total of 16 RDs generated positive signals across all four algorithms (Table 2). Among these, antiallergics accounted for the majority of signals (12/16, 75.00%), comprising one leukotriene receptor antagonist (LTRA) and eleven antihistamines. The remaining signals included a cystic fibrosis transmembrane conductance regulator (CFTR) protein potentiator, a central nervous system (CNS) stimulant, a β2 receptor agonist, and an antimicrobial agent.

**Table 2:** Identification of RD-related pAE signals using disproportionality analysis in pediatric patients

Drugs	Reports	ROR (95% Cl)	PRR (95% Cl)	MHRA PRR (χ^2^)	BCPNN IC (IC025)
Montelukast	6,168	11.37 (11.04, 11.71)	9.15 (8.94, 9.37)	9.15 (40,612.50)	3.04 (2.99)
Promethazine	308	10.74 (9.46, 12.19)	8.57 (7.77, 9.46)	8.57 (2103.12)	3.09 (2.87)
Elexacaftor-ivacaftor-tezacaftor	287	2.96 (2.62, 3.34)	2.81 (2.52, 3.15)	2.81 (342.92)	1.49 (1.30)
Diphenhydramine	278	2.13 (1.89, 2.41)	2.07 (1.85, 2.32)	2.07 (157.09)	1.05 (0.86)
Desloratadine	74	2.55 (2.01, 3.23)	2.45 (1.97, 3.06)	2.45 (65.15)	1.29 (0.92)
Levocetirizine	73	2.18 (1.72, 2.76)	2.12 (1.69, 2.65)	2.12 (44.06)	1.08 (0.71)
Hydroxyzine	53	2.13 (1.61, 2.81)	2.07 (1.59, 2.69)	2.07 (29.93)	1.05 (0.61)
Loratadine-pseudoephedrine	19	2.15 (1.35, 3.42)	2.09 (1.35, 3.23)	2.09 (11.06)	1.06 (0.32)
Cyproheptadine	14	2.22 (1.29, 3.80)	2.15 (1.29, 3.57)	2.15 (8.82)	1.10 (0.22)
Doxylamine	14	2.40 (1.40, 4.12)	2.31 (1.39, 3.85)	2.31 (10.73)	1.21 (0.32)
Promethazine-thiourea	9	37.36 (14.83, 94.11)	19.18 (12.08, 30.44)	19.18 (159.20)	4.26 (1.61)
Caffeine	8	2.64 (1.29, 5.42)	2.54 (1.30, 4.95)	2.54 (7.64)	1.34 (0.11)
Terbutaline	8	2.82 (1.37, 5.78)	2.69 (1.38, 5.25)	2.69 (8.73)	1.43 (0.17)
Ebastine	6	4.77 (2.04, 11.15)	4.34 (2.04, 9.22)	4.34 (15.84)	2.12 (0.38)
Dextromethorphan-promethazine	4	10.67 (3.51, 32.42)	8.52 (3.59, 20.23)	8.52 (27.27)	3.09 (0.29)
Oxytetracycline	4	8.79 (2.96, 26.12)	7.31 (3.02, 17.64)	7.31 (22.35)	2.87 (0.24)

Abbreviations: *RD* respiratory drug, *pAE* psychiatric adverse event, *ROR* reporting odds ratio, *CI* confidence interval, *PRR* proportional reporting ratio, *MHRA* Medicines and Healthcare products Regulatory Agency, *BCPNN* Bayesian confidence propagation neural network, *IC* information component

Montelukast was the most frequently reported drug (*n* = 6,168), demonstrating strong disproportionality evidence (ROR = 11.37; PRR = 9.15; χ^2^ = 40,612.50; information component [IC] = 3.04). Promethazine followed as the second most reported drug (*n* = 308) with notable signal strength (ROR = 10.74, PRR = 8.57, χ^2^ = 2,103.12, IC = 3.09). Elexacaftor-ivacaftor-tezacaftor also showed significant disproportionality signals (*n* = 287, ROR = 2.96, PRR = 2.81, χ^2^ = 342.92, IC = 1.49). Of particular note, promethazine-thiourea displayed the highest signal strength (ROR = 37.36), despite being associated with a relatively small number of reports (*n* = 9).

###  PT level analysis

We further analyzed the specific PTs associated with RD-related pAEs (Table 3). Montelukast was predominantly associated with anxiety (*n* = 1,347, 21.84%, ROR = 15.08), depression (*n* = 978, 15.86%, ROR = 12.90), and suicidal ideation (*n* = 880, 14.27%, ROR = 13.63). Promethazine demonstrated the strongest signal for intentional self-injury (*n* = 194, 62.99%, ROR = 127.36) and akathisia (*n* = 31, 10.06%, ROR = 89.46). For elexacaftor-ivacaftor-tezacaftor, the most frequently reported pAEs were anxiety (*n* = 74, 25.78%) and depression (*n* = 54, 18.82%). Diphenhydramine (ROR = 15.92) and hydroxyzine (ROR = 20.45) exhibited concerning signals for completed suicide.

**Table 3:** Characteristics of main PTs from RD-related pAE reports in pediatric patients

Drug^#^	PTs	N (%)	ROR (95% CI)
Montelukast (*n* = 6,168)	Anxiety	1,347 (21.84)	15.08 (14.20, 16.01)
	Depression	978 (15.86)	12.90 (12.04, 13.82)
	Suicidal ideation	880 (14.27)	13.63 (12.67, 14.67)
	Agitation	357 (5.79)	4.43 (3.98, 4.94)
	Depressed mood	350 (5.67)	14.67 (13.06, 16.47)
Promethazine (*n* = 308)	Intentional self-injury	194 (62.99)	127.36 (108.90, 148.95)
	Akathisia	31 (10.06)	89.46 (62.02, 129.04)
	Anxiety	29 (9.42)	5.54 (3.83, 8.01)
	Agitation	25 (8.12)	6.23 (4.19, 9.26)
	Suicide attempt	10 (3.25)	2.61 (1.40, 4.86)
Elexacaftor-ivacaftor-tezacaftor (*n* = 287)	Anxiety	74 (25.78)	5.00 (3.97, 6.30)
	Depression	54 (18.82)	4.43 (3.39, 5.81)
	Suicidal ideation	32 (11.15)	3.04 (2.15, 4.32)
	Mental disorder	20 (6.97)	8.59 (5.52, 13.38)
	Depressed mood	20 (6.97)	5.17 (3.33, 8.04)
Diphenhydramine (*n* = 278)	Completed suicide	100 (35.97)	15.92 (13.01, 19.48)
	Agitation	78 (28.06)	5.23 (4.18, 6.55)
	Suicide attempt	38 (13.67)	2.67 (1.94, 3.67)
Desloratadine (*n* = 74)	Agitation	14 (18.92)	4.13 (2.44, 7.01)
	Suicide attempt	13 (17.57)	4.07 (2.35, 7.04)
	Intentional self-injury	9 (12.16)	5.71 (2.96, 11.02)
Levocetirizine (*n* = 73)	Suicide attempt	14 (19.18)	3.83 (2.26, 6.50)
	Depression	13 (17.81)	3.14 (1.81, 5.42)
	Suicidal ideation	8 (10.96)	2.24 (1.12, 4.50)
Hydroxyzine (*n* = 53)	Completed suicide	25 (47.17)	20.45 (13.72, 30.48)
	Suicide attempt	10 (18.87)	3.68 (1.97, 6.87)
	Agitation	7 (13.21)	2.42 (1.15, 5.09)
Loratadine-pseudoephedrine (*n* = 19)	Agitation	6 (31.58)	5.91 (2.64, 13.25)
	Anxiety	4 (21.05)	3.00 (1.12, 8.03)
Cyproheptadine (*n* = 14)	Agitation	10 (71.43)	14.09 (7.48, 26.53)
Doxylamine (*n* = 14)	Agitation	4 (28.57)	5.93 (2.20, 15.93)
	Suicide attempt	3 (21.43)	4.69 (1.50, 14.67)
	Depression	3 (21.43)	4.14 (1.32, 12.93)

^#^: Only sufficient reports (i.e., > 10) were included in the final analysis

Abbreviations: *PT* Preferred term, *RD* Respiratory drug, *pAE* Psychiatric adverse event, *ROR* Reporting odds ratio, *CI* Confidence interval

**Fig. 2:**
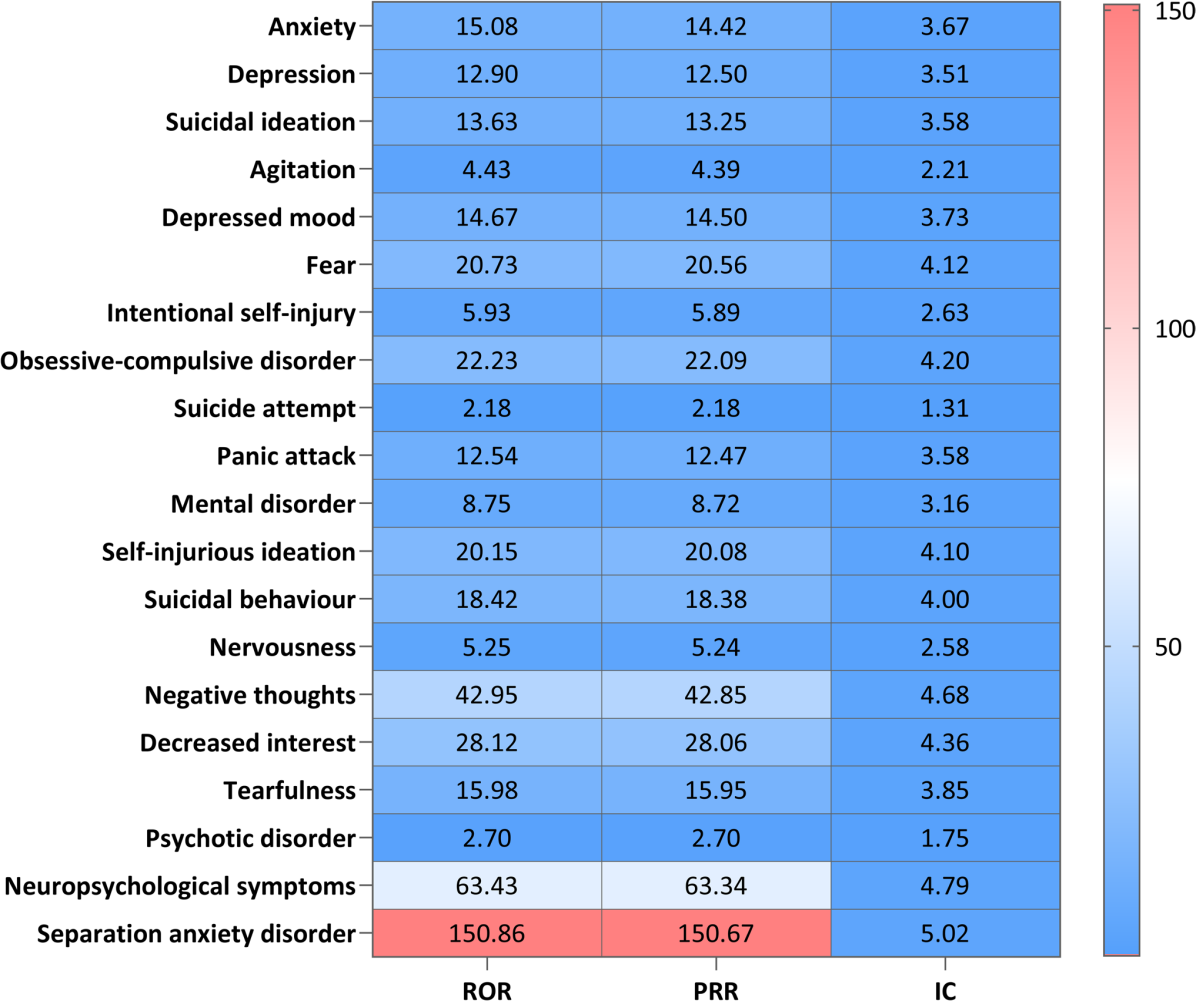
PT level analysis of RD-related pAE risk for montelukast. Abbreviation: PT, preferred term; RD, respiratory drug; pAE, psychiatric adverse event; ROR, reporting odds ratio; PRR, proportional reporting ratio; IC, information component

Given montelukast's predominance in the dataset, a detailed disproportionality analysis was conducted for its associated pAEs (Fig. 2). Notably, separation anxiety disorder exhibited the strongest signal (ROR = 150.86). Other significant signals included neuropsychological symptoms (ROR = 63.43) and negative thoughts (ROR = 42.95).

###  Focused HLGT analysis

**Fig. 3:**
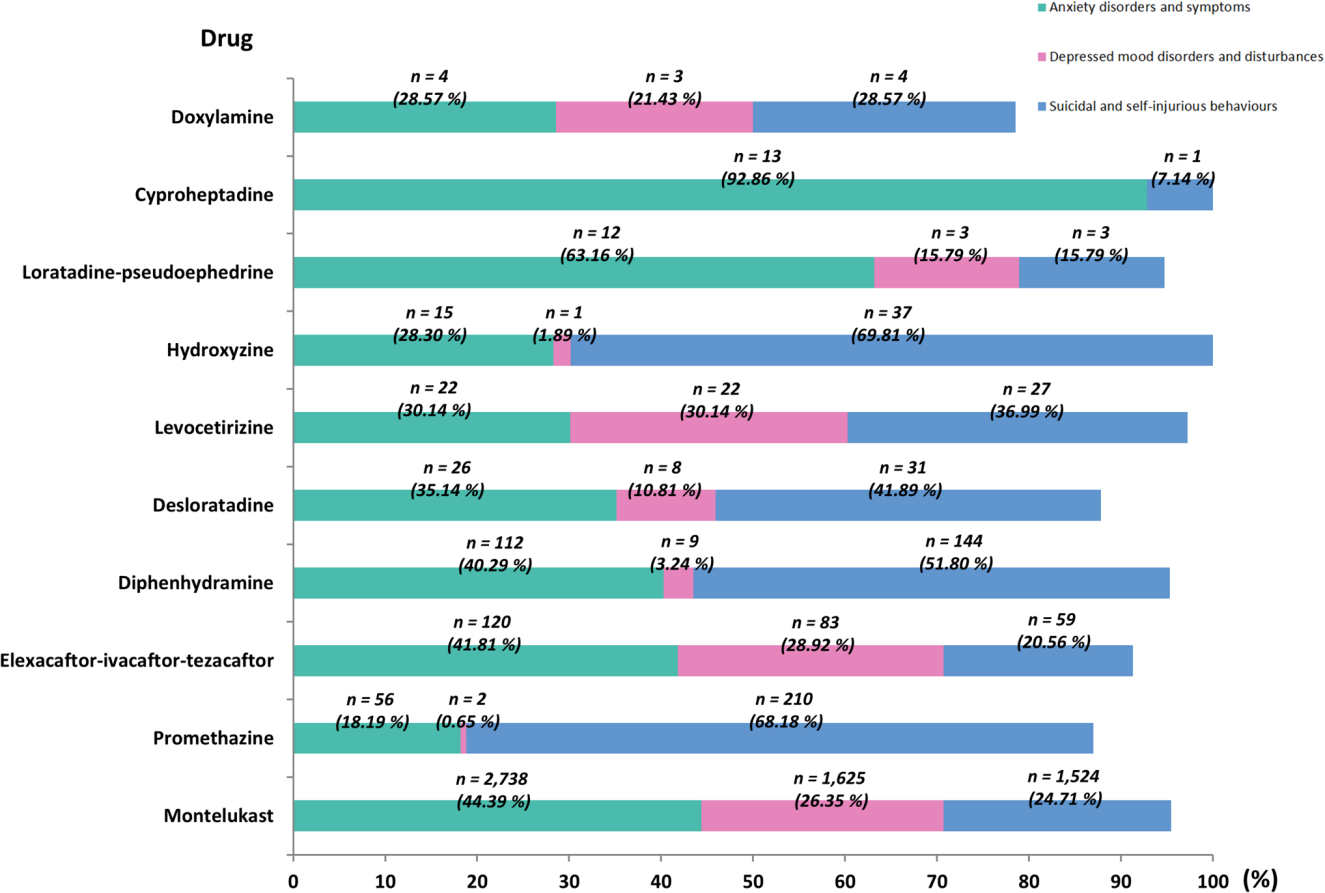
Characteristics of focused HLGTs from RD-related pAE reports in pediatric patients^#^. #: Only sufficient reports (i.e., > 10) were included in the final analysis. Abbreviation: HLGT, high level group term; RD, respiratory drug; pAE, psychiatric adverse event

RD-related pAE reports were analyzed at the HLGT level, focusing on three major psychiatric categories: anxiety disorders and symptoms, depressive mood disorders and disturbances, and suicidal and self-injurious behaviors (Table S3, Fig. 3). Among the ten drugs with sufficient reporting data (i.e., > 10 reports), montelukast exhibited a relatively balanced distribution of psychiatric disorders. Anxiety disorders and symptoms were the most frequently reported (*n* = 2,738, 44.39%), followed by depressed mood disorders and disturbances (*n* = 1,625, 26.35%) and suicidal and self-injurious behaviors (*n* = 1,524, 24.71%). In contrast, promethazine demonstrated a strong association with suicidality, with suicidal and self-injurious behaviors accounting for the majority of reports (n = 210, 68.18%), while reports of depressive disorders were minimal (*n* = 2, 0.65%). Similarly, hydroxyzine showed a pronounced association with suicidality (*n* = 37, 69.81%). Elexacaftor-ivacaftor-tezacaftor exhibited a pattern similar to montelukast, with anxiety predominating (*n* = 120, 41.81%). Additionally, cyproheptadine demonstrated the highest risk of anxiety among the evaluated drugs, with anxiety-related reports comprising 92.86% (*n* = 13) of its total psychiatric pAE reports.

###  Serious outcomes of death and life-threatening events

**Fig. 4:**
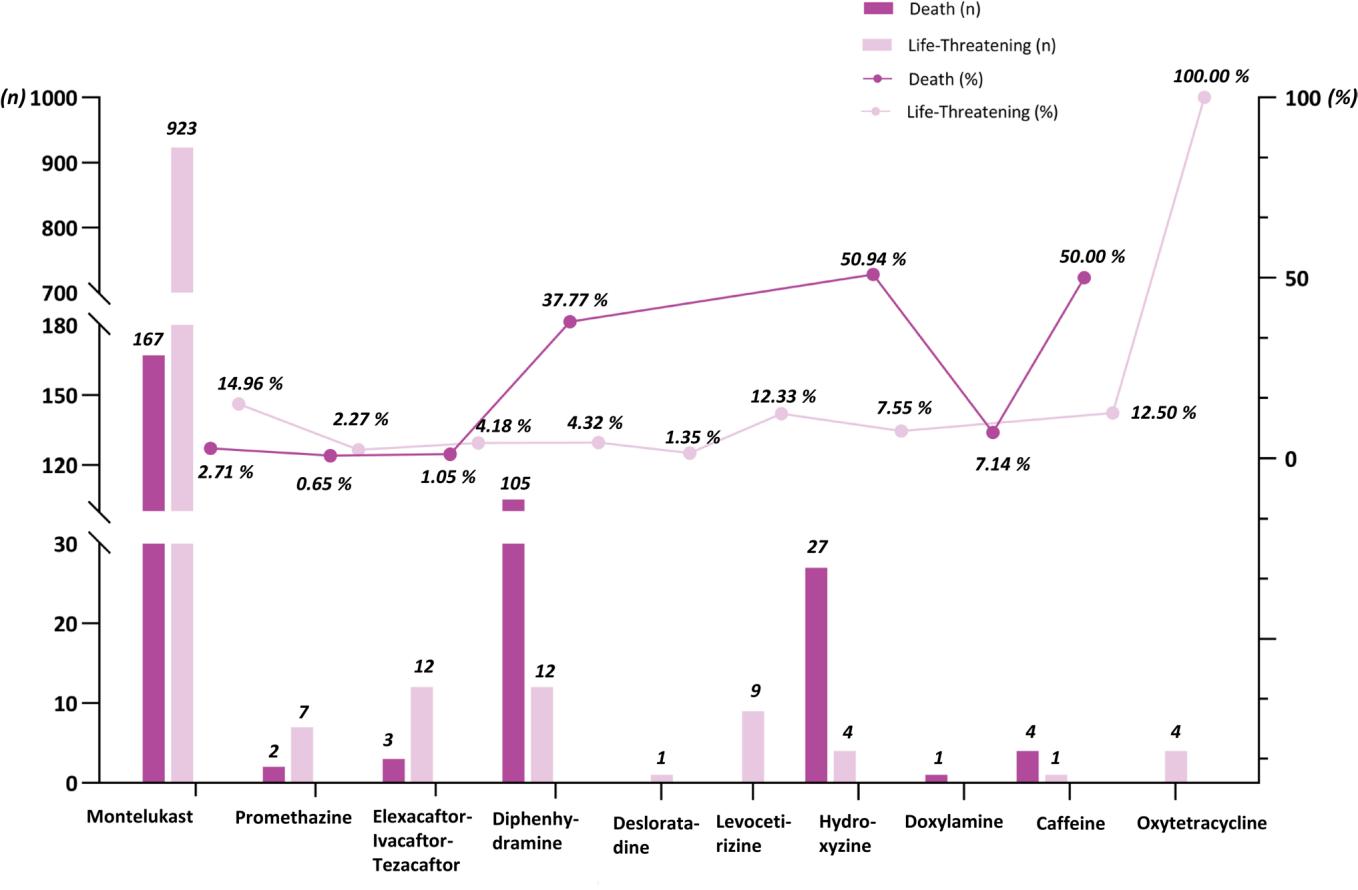
Proportion of death and life-threatening events in RD-related pAE reports. Note: Of the remaining 6 drugs (loratadine-pseudoephedrine, cyproheptadine, promethazine-thiourea, terbutaline, ebastine, and dextromethorphan-promethazine) among the 16 respiratory drugs, no deaths or life-threatening events have been reported. Abbreviation: RD, respiratory drug; pAE, psychiatric adverse event

We analyzed serious outcomes from RD-related pAE reports, focusing on deaths and life-threatening events across different drugs (Table S4, Fig. 4). Montelukast accounted for the highest absolute number of deaths (n = 167) and life-threatening events (n = 923). However, the highest death rates among RD-related pAEs were observed for hydroxyzine (27/53, 50.94%), followed by caffeine (4/8, 50.00%), and diphenhydramine (105/278, 37.77%). In terms of life-threatening events, the highest proportions were seen with oxytetracycline (4/4, 100.00%), followed by montelukast (923/6,168, 14.96%).

###  Stratified analysis

**Fig. 5:**
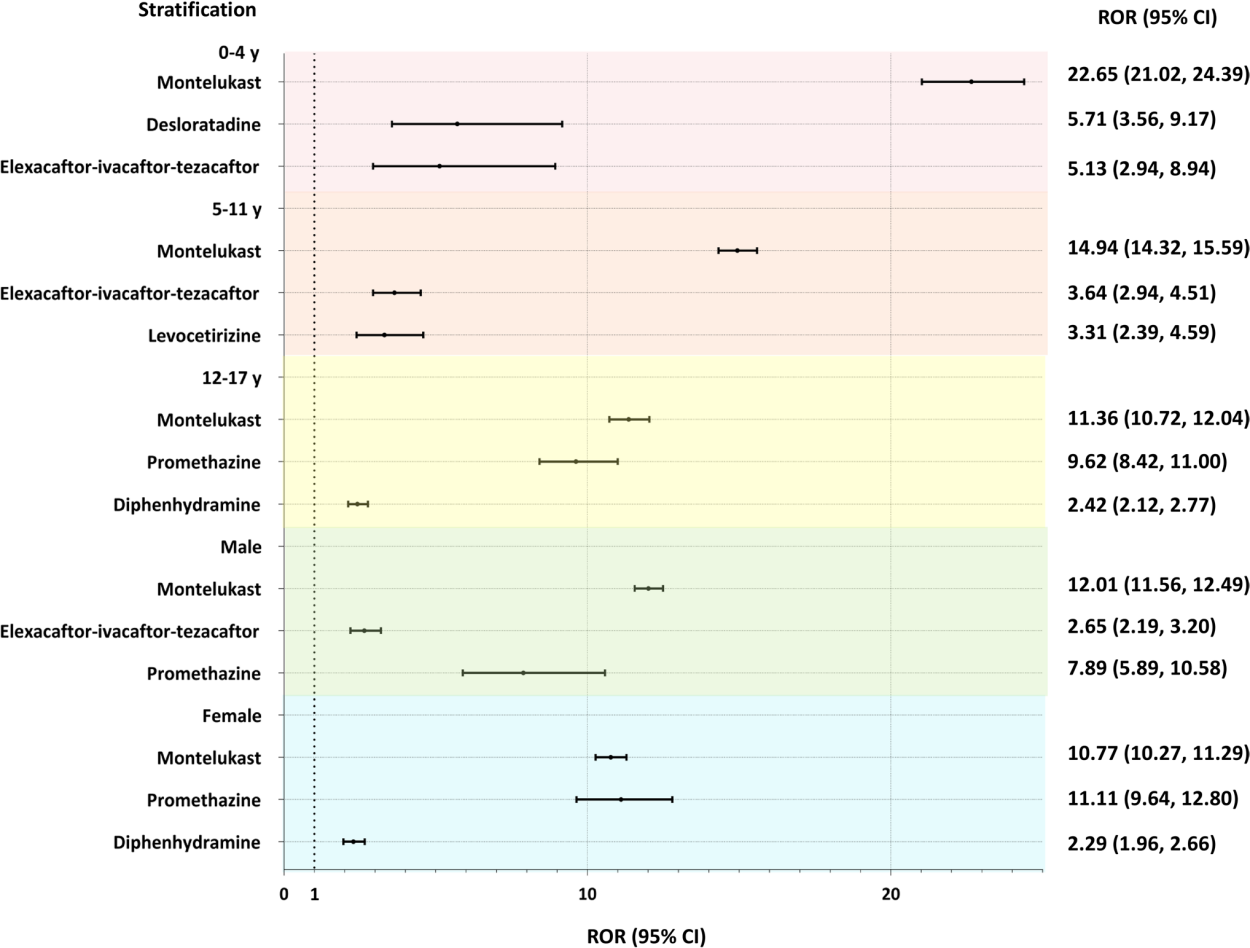
Forest plot depicting the stratified analysis of RD-related pAE reports by age and gender. Abbreviation: RD, respiratory drug; pAE, psychiatric adverse event; ROR, reporting odds ratio; CI, confidence interval

To further characterize RD-related pAE signals, stratified analyses were conducted by age and gender (Table S5, Fig. 5). Across all age groups, montelukast accounted for the highest number of reports, with the strongest disproportionality signal observed in younger children (0–4 years: ROR = 22.65). In this age group, desloratadine (ROR = 5.71) and elexacaftor-ivacaftor-tezacaftor (ROR = 5.13) ranked second and third, respectively. For children aged 5–11 years, montelukast remained the most frequently reported drug (ROR = 14.94), followed by elexacaftor-ivacaftor-tezacaftor (ROR = 3.64) and levocetirizine (ROR = 3.31). In adolescents aged 12–17 years, montelukast continued to demonstrate strong disproportionality signals (ROR = 11.36), followed by promethazine (ROR = 9.62) and diphenhydramine (ROR = 2.42).

Gender-stratified analysis revealed that montelukast generated stronger signals in males (ROR = 12.01) compared to females (ROR = 10.77). Conversely, promethazine exhibited higher signals in females (ROR = 11.11) than in males (ROR = 7.89).

## Discussion

Using a comprehensive analysis of the FAERS database spanning two decades, sixteen RDs were identified to have pAE signals across all four disproportionality algorithms. Among these, antiallergics represented the majority, with montelukast emerging as the most frequently reported (n = 6,168). Strong disproportionality signals were observed for promethazine-thiourea (ROR = 37.36), montelukast (ROR = 11.37), and promethazine (ROR = 10.74). Of particular concern were signals for completed suicide associated with diphenhydramine (ROR = 15.92) and hydroxyzine (ROR = 20.45), as well as suicidal ideation linked to montelukast (ROR = 13.63). Stratified analyses revealed age- and gender-specific differences in reporting patterns.

In the analysis of antihistamines, first-generation antihistamines (e.g., promethazine, diphenhydramine, and hydroxyzine) demonstrated strong signals for pAEs. At the HLGT level, hydroxyzine and promethazine were predominantly associated with suicidal and self-injurious behaviors, while second-generation antihistamines (e.g., desloratadine and levocetirizine) exhibited more balanced distributions across anxiety disorders and symptoms and suicidal and self-injurious behaviors. These discrepancies may reflect the pharmacological differences between first- and second-generation antihistamines (Simons 2010), particularly their blood–brain barrier (BBB) penetration abilities. Compared to second-generation agents, which are more selective for peripheral H1 receptors, first-generation antihistamines can readily cross the BBB and exhibit broader receptor activities (Parisi et al. 2020). These findings have implications for the selection of antihistamines in pediatric clinical practice. First-generation antihistamines should be used cautiously in pediatric patients, particularly adolescents, due to their strong signals for suicidal behavior. In contrast, suicidal behavior associated with desloratadine and levocetirizine has been relatively rare. When antihistamines are necessary, second-generation agents may be preferable from a neuropsychiatric safety perspective (Fein et al. 2019).

Montelukast, a LTRA widely used for the management of asthma and allergic rhinitis, is approved for use in children aged one year and older (2021). In March 2020, the FDA issued a black box warning to highlight the potential for severe neuropsychiatric adverse events associated with montelukast use (U.S. Food and Drug Administration 2020). In this study, montelukast accounted for the highest number of reports associated with pAEs (88.2%, 6,168/6,994). At the PT level, the most frequently reported psychiatric events for montelukast were anxiety (21.84%), depression (15.86%), and suicidal ideation (14.27%), findings consistent with the HLGT analysis. Notably, the strongest signal was observed for separation anxiety disorder (ROR = 150.86), underscoring the severity of pAEs associated with montelukast.

The relationship between montelukast use and psychiatric events in children has been inconsistently reported. A cohort study reported an increased incidence of neuropsychiatric adverse reactions when montelukast was used as an adjunct to inhaled corticosteroids (ICS) in children with asthma (Paljarvi et al. 2024). Similarly, a systematic review described the risk of neuropsychiatric events associated with montelukast in both pediatric and adult asthma patients (Lo et al. 2023). However, a recent cohort study suggested no association between montelukast use and the risk of neuropsychiatric adverse events in children (Wintzell et al. 2025). These conflicting findings highlight the need for further research to establish a definitive relationship between montelukast use and pAEs.

The exact mechanism underlying psychiatric adverse events reported with montelukast use remains unclear. The FDA once updated the prescribing information for montelukast (2021), acknowledging that earlier clinical trials underestimated the drug's ability to cross the BBB, which is now recognized to occur at significantly higher levels than previously reported. Studies in young mouse models have demonstrated that montelukast exerts direct effects on the brain, including reduced neuronal proliferation in the hippocampus (Eriksson et al. 2018). An integrated metabolomics and proteomics analysis explored the neuropsychiatric effects of montelukast and is associated with changes in the hypothalamic–pituitary–adrenal (HPA) axis and brain oxidative state (Marques et al. 2022). Dysregulation of the HPA axis during childhood has the potential to alter neurodevelopment, thereby increasing the risk of psychiatric disorders (Tottenham and Sheridan 2009).

Our identification of psychiatric signals for elexacaftor-ivacaftor-tezacaftor (ROR = 2.96), a CFTR modulator used in cystic fibrosis, represents a novel clinical finding. Its psychiatric profile closely resembled that of montelukast, with predominant symptoms of anxiety (41.81%) and depression (28.92%). This is particularly noteworthy, as previous summaries of product characteristic (SPC) for elexacaftor-ivacaftor-tezacaftor did not report related pAEs (2024). More recently, potential neuropsychiatric adverse event signals associated with CFTR modulators have also been reported in other studies (McKinzie et al. 2017; Ibrahim et al. 2023; Nidegger et al. 2025), findings consistent with our research. While the exact mechanism remains unclear, these results underscore the clinical importance of counseling and monitoring patients for psychiatric effects during CFTR modulator therapy (Talwalkar et al. 2017).

Our study highlights the important implications of serious outcomes and onset timing for clinical practice. Serious outcomes were reported in 82.59% of all RD-related pAE cases. Of particular concern were the high mortality rates associated with hydroxyzine (50.94%), caffeine (50.00%), and diphenhydramine (37.77%). While montelukast had a relatively lower mortality rate (2.71%), it accounted for the highest absolute number of deaths (*n* = 167) and life-threatening events (*n* = 923). These findings suggest that while montelukast-related pAEs may be more frequent, first-generation antihistamines and CNS stimulants may pose a greater risk for fatal outcomes when adverse psychiatric events occur. Besides, 27.87% of RD-related pAE cases occurred within the first week of treatment. However, over half of the reported cases (55.42%) occurred between 181–360 days of treatment. This highlights the necessity of continued vigilance throughout the treatment course, even during long-term therapy.

Age-stratified analysis revealed significant age-dependent variations in RD-related pAE reports. Montelukast demonstrated the highest signal strength among younger children (0–4 years: ROR = 22.65), followed by school-age children (5–11 years: ROR = 14.94) and adolescents (12–17 years: ROR = 11.36). These differences may reflect developmental variations in neurotransmitter systems and BBB permeability (Marques et al. 2022). In contrast, promethazine (ROR = 9.62) and diphenhydramine (ROR = 2.42) were more prominent in adolescents. This could be related to pharmacokinetic differences in this age group or an increased propensity for risk-taking behaviors during adolescence.

Montelukast showed slightly higher signal strength in males (ROR = 12.01) compared to females (ROR = 10.77). Conversely, promethazine demonstrated higher signal strength in females (ROR = 11.11) than in males (ROR = 7.89). These observations may reflect age- and gender-related differences in reporting patterns, potentially influenced by differences in drug metabolism or receptor sensitivity (Ferrajolo et al. 2019).

This study has several limitations: First, as a pharmacovigilance study based on spontaneous reporting data, inherent reporting biases exist, including underreporting of less severe events, selective reporting of unexpected events, and stimulated reporting following media attention or regulatory changes. Second, the variability in reporting can be influenced by geographic region, healthcare settings, and time periods. Third, reporting is influenced by diverse factors such as reporter awareness, seriousness of reactions, and regulatory actions, resulting in potential biases of the true adverse event pattern. Fourth, Due to the absence of denominators, we were unable to calculate the incidence of RD-related pAEs or estimate related risks. Fifth, causality cannot be established based on disproportionality analysis alone. Additionally, psychiatric symptoms might be manifestations of underlying respiratory conditions rather than drug effects. Finally, as an exploratory study, our findings require validation through prospective clinical trials.

## Conclusion

This pharmacovigilance study provides comprehensive evidence on the spectrum of psychiatric disorders associated with RDs in pediatric populations. Sixteen RDs with pAE signals were identified using disproportionality algorithms. Montelukast and first-generation antihistamines demonstrated strong signals for psychiatric-related adverse events, with notable age- and gender-specific susceptibilities. The proportion of serious outcomes was extremely high, including deaths and life-threatening events. These findings underscore the importance of strengthening the monitoring of psychiatric symptoms throughout the course of treatment, optimizing clinical decision-making processes, and fostering interdisciplinary collaboration. Such efforts can provide robust scientific evidence to support global public health initiatives, ultimately improving the safety of respiratory drug use in pediatric populations.

## Supplementary Information

Below is the link to the electronic supplementary material.

Supplementary file1 (DOCX 55 KB)

## Data Availability

All source data for this work (or generated in this study) are available upon reasonable request.
