# Supplementary material for: Respiratory drugs and psychiatric adverse events in children and adolescents: a pharmacovigilance study based on the FAERS database
Source: Naunyn Schmiedebergs Arch Pharmacol. 2026 Feb 6;399(7):10535–47. doi: 10.1007/s00210-026-05075-5 (PMC13152991; doi:10.1007/s00210-026-05075-5)
Supplement: Supplementary file 1 — Supplementary file1 (DOCX 55 KB) [file 210_2026_5075_MOESM1_ESM.docx]

**SUPPLEMENTARY MATERIALS**

**Tables**

Table S1 2 × 2 contingency table for disproportionality analysis

Table S2 ROR, PRR, MHRA and BCPNN methods, formulas, and thresholds

Table S3 Characteristics of focused HLGTs from RD-related pAE reports in pediatric patients

Table S4 Proportion of death and life-threatening events in RD-related pAE reports

Table S5 Stratified analysis of RD-related pAE reports by age and gender based on the number of cases

**Table S1 2 × 2 contingency table for disproportionality analysis**

|  | **Target pAEs reported** | **Other AEs reported** | **Total** |
| --- | --- | --- | --- |
| Target respiratory drug | a | b | a+b |
| Other drugs | c | d | c+d |
| Total | a+c | b+d | a+b+c+d |

a = The number of reports of drug with the adverse event of interest.

b = The number of reports of all other drugs with the adverse event of interest.

c = The number of reports of drugs with all other adverse events.

d = The number of reports of all other drugs with all other adverse events.

Abbreviations: AE, adverse event; pAE, psychiatric adverse event.

**Table S2 ROR, PRR, MHRA, and BCPNN methods, formulas, and thresholds**

| **Method** | **Calculation formula** | **Threshold** |
| --- | --- | --- |
| ROR | $ROR=\frac{\text{（}a/c\text{）}}{(b/d)}=\frac{ad}{bc}$ $SE\text{（}1\text{n}ROR\text{）}=\sqrt{(\frac{1}{a}+\frac{1}{b}+\frac{1}{c}+\frac{1}{\text{d}})}$    95% CI=*e*^1n(ROR)±1.96SE^ | a ≥ 3  95%CI (lower limit) > 1 |
| PRR | $PRR=\frac{\text{a}/\text{（}a+b\text{）}}{c/(c+d)}$ $SE\text{（}1\text{n}PRR\text{）}=\sqrt{(\frac{1}{\text{a}}-\frac{1}{a+b}+\frac{1}{c}-\frac{1}{c+d})}$    95% CI=*e*^1n(PRR)±1.96SE^ | a ≥ 3  95%CI (lower limit) > 1 |
| MHRA | $\chi2 =\frac{{(ad-bc)}^{2}(a+b+c+d)}{( a+b)(a+c)(c+d)(b+d)}$ | a ≥ 3  PRR ≥ 2  $\chi2\geq4$ |
| BCPNN | $IC=\log_{2}\frac{p(x,y)}{p(x)p(y)}$=  E(IC)=${log}_{2}\frac{(a+\gamma11)(a+b+c+d+\alpha)(a+b+c+d+\beta)}{（a+b+c+d+\gamma）(a+b+\alpha1)(a+c+\beta1)}$  V(IC)=$\frac{1}{\text{(l}n2)^{2}}\left\{\left[\frac{(a+b+c+d-a+\gamma-\gamma11)}{(a+\gamma11)(1+a+b+c+d+\gamma)}\right]+\left[\frac{(a+b+c+d)-(a+b)+\alpha-\alpha1}{(a+b+\alpha1)(1+a+b+c+d+\alpha)}\right]+\left[\frac{(a+b+c+d)-(a+c)+\beta-\beta1}{(a+c+\beta1)(1+a+b+c+d+\beta)}\right]\right\}$ $\gamma=\gamma11\frac{(a+b+c+d+\alpha)(a+b+c+d+\beta)}{(a+b+\alpha1)(a+c+\beta1)}$ $IC-2SD=E(IC)-2\sqrt{V(IC)}$     | IC025 > 0 |

Equation: a = The number of reports of drug with the adverse event of interest.

b = The number of reports of all other drugs with the adverse event of interest.

c = The number of reports of drug with all other adverse events.

d = The number of reports of all other drugs with all other adverse events.

Abbreviations: ROR, reporting odds ratio; PRR, proportional reporting ratio; MHRA, Medicines and Healthcare products Regulatory Agency; BCPNN, Bayesian confidence propagation neural network; CI, confidence interval; χ2, chi-squared; IC, information component; IC025, the lower limit of the 95% one-sided CI, of the IC.

**Table S3 Characteristics of focused HLGTs from RD-related pAE reports in pediatric patients**

| Drug | Reports | Focused HLGTs | n | % |
| --- | --- | --- | --- | --- |
| Montelukast | 6,168 | Anxiety disorders and symptoms | 2,738 | 44.39 |
|  |  | Depressed mood disorders and disturbances | 1,625 | 26.35 |
|  |  | Suicidal and self-injurious behaviors | 1,524 | 24.71 |
| Promethazine | 308 | Anxiety disorders and symptoms | 56 | 18.19 |
|  |  | Depressed mood disorders and disturbances | 2 | 0.65 |
|  |  | Suicidal and self-injurious behaviors | 210 | 68.18 |
| Elexacaftor-ivacaftor-tezacaftor | 287 | Anxiety disorders and symptoms | 120 | 41.81 |
|  |  | Depressed mood disorders and disturbances | 83 | 28.92 |
|  |  | Suicidal and self-injurious behaviors | 59 | 20.56 |
| Diphenhydramine | 278 | Anxiety disorders and symptoms | 112 | 40.29 |
|  |  | Depressed mood disorders and disturbances | 9 | 3.24 |
|  |  | Suicidal and self-injurious behaviors | 144 | 51.8 |
| Desloratadine | 74 | Anxiety disorders and symptoms | 26 | 35.14 |
|  |  | Depressed mood disorders and disturbances | 8 | 10.81 |
|  |  | Suicidal and self-injurious behaviors | 31 | 41.89 |
| Levocetirizine | 73 | Anxiety disorders and symptoms | 22 | 30.14 |
|  |  | Depressed mood disorders and disturbances | 22 | 30.14 |
|  |  | Suicidal and self-injurious behaviors | 27 | 36.99 |
| Hydroxyzine | 53 | Anxiety disorders and symptoms | 15 | 28.30 |
|  |  | Depressed mood disorders and disturbances | 1 | 1.89 |
|  |  | Suicidal and self-injurious behaviors | 37 | 69.81 |
| Loratadine-pseudoephedrine | 19 | Anxiety disorders and symptoms | 12 | 63.16 |
|  |  | Depressed mood disorders and disturbances | 3 | 15.79 |
|  |  | Suicidal and self-injurious behaviors | 3 | 15.79 |
| Cyproheptadine | 14 | Anxiety disorders and symptoms | 13 | 92.86 |
|  |  | Depressed mood disorders and disturbances | 0 | 0 |
|  |  | Suicidal and self-injurious behaviors | 1 | 7.14 |
| Doxylamine | 14 | Anxiety disorders and symptoms | 4 | 28.57 |
|  |  | Depressed mood disorders and disturbances | 3 | 21.43 |
|  |  | Suicidal and self-injurious behaviors | 4 | 28.57 |

Note: If the total reports were less than 10, it was excluded from the analysis.

Abbreviations: HLGT, high level group term; RD, respiratory drugs; pAE, psychiatric adverse event.

**Table S4 Proportion of death and life-threatening events in RD-related pAE reports**

| Drugs | Total (n) | Death (n, %) | Life-Threatening (n, %) |
| --- | --- | --- | --- |
| Montelukast | 6,168 | 167 (2.71) | 923 (14.96) |
| Promethazine | 308 | 2 (0.65) | 7 (2.27) |
| Elexacaftor-Ivacaftor-Tezacaftor | 287 | 3 (1.05) | 12 (4.18) |
| Diphenhydramine | 278 | 105 (37.77) | 12 (4.32) |
| Desloratadine | 74 | 0 (0) | 1 (1.35) |
| Levocetirizine | 73 | 0 (0) | 9 (12.33) |
| Hydroxyzine | 53 | 27 (50.94) | 4 (7.55) |
| Doxylamine | 14 | 1 (7.14) | 0 (0) |
| Caffeine | 8 | 4 (50.00) | 1 (12.50) |
| Oxytetracycline | 4 | 0 (0) | 4 (100.00) |

Note: Of the remaining 6 drugs (loratadine-pseudoephedrine, cyproheptadine, promethazine-thiourea, terbutaline, ebastine, and dextromethorphan-promethazine) among the 16 respiratory drugs, no deaths or life-threatening events have been reported.

Abbreviations: RD, respiratory drugs; pAE, psychiatric adverse event.

**Table S5 Stratified analysis of RD-related pAE reports by age and gender based on the number of cases**

| Stratification | Drug | Reports | ROR  (95% CI) | PRR  (95% CI) | MHRA  (χ2) | BCPNN  (IC025) |
| --- | --- | --- | --- | --- | --- | --- |
| 0-4 y | Montelukast | 997 | 22.65 (21.02, 24.39) | 19.90 (18.61, 21.27) | 19.90 (13998.5) | 3.97 (3.84) |
|  | Desloratadine | 18 | 5.71 (3.56, 9.17) | 5.50 (3.50, 8.65) | 5.50 (66.61) | 2.46 (1.47) |
|  | Elexacaftor-ivacaftor-tezacaftor | 13 | 5.13 (2.94, 8.94) | 4.96 (2.91, 8.46) | 4.96 (41.36) | 2.31 (1.16) |
| 5-11 y | Montelukast | 3,475 | 14.94 (14.32, 15.59) | 11.78 (11.38, 12.19) | 11.78 (26507.4) | 3.19 (3.13) |
|  | Elexacaftor-ivacaftor-tezacaftor | 93 | 3.64 (2.94, 4.51) | 3.42 (2.81, 4.15) | 3.42 (162.10) | 1.77 (1.42) |
|  | Levocetirizine | 39 | 3.31 (2.39, 4.59) | 3.13 (2.31, 4.23) | 3.13 (57.80) | 1.64 (1.09) |
| 12-17 y | Montelukast | 1,696 | 11.36 (10.72, 12.04) | 8.27 (7.94, 8.62) | 8.27 (10709.2) | 2.98 (2.90) |
|  | Promethazine | 298 | 9.62 (8.42, 11.00) | 7.27 (6.60, 8.02) | 7.27 (1661.41) | 2.85 (2.63) |
|  | Diphenhydramine | 232 | 2.42 (2.12, 2.77) | 2.30 (2.03, 2.60) | 2.30 (176.18) | 1.20 (0.99) |
| Male | Montelukast | 3,765 | 12.01 (11.56, 12.49) | 9.68 (9.38, 9.98) | 9.68 (25584.4) | 3.07 (3.01) |
|  | Elexacaftor-ivacaftor-tezacaftor | 116 | 2.65 (2.19, 3.20) | 2.54 (2.13, 3.03) | 2.54 (110.94) | 1.34 (1.05) |
|  | Promethazine | 54 | 7.89 (5.89, 10.58) | 6.72 (5.27, 8.57) | 6.72 (269.23) | 2.75 (2.18) |
| Female | Montelukast | 2,351 | 10.77 (10.27, 11.29) | 8.62 (8.30, 8.96) | 8.62 (14846.0) | 2.99 (2.92) |
|  | Promethazine | 252 | 11.11 (9.64, 12.80) | 8.70 (7.81, 9.70) | 8.70 (1749.45) | 3.11 (2.86) |
|  | Diphenhydramine | 178 | 2.29 (1.96, 2.66) | 2.21 (1.91, 2.55) | 2.21 (120.25) | 1.14 (0.90) |

Abbreviation: RD, respiratory drug; pAE, psychiatric adverse event; ROR, reporting odds ratio; CI, confidence interval; PRR, rroportional reporting ratio; MHRA, Medicines and Healthcare products Regulatory Agency; BCPNN, Bayesian confidence propagation neural network; IC, information component.
